# Evaluation of the relationship between computed tomography angiography collateral scores and clinical outcome

**DOI:** 10.1055/s-0044-1779268

**Published:** 2024-03-04

**Authors:** Ahmet Yabalak, Muhammed Nur Ögün, Ayşenur Önalan, Murat Yılmaz, Hilmiye Tokmak, Sadettin Ersoy, Fatma Bilgili, Tahsin Bakkal

**Affiliations:** 1 Duzce University, Faculty of Medicine, Department of Neurology, Duzce, Türkiye.; 2 Bolu Abant Izzet Baysal University, Department of Neurology, Bolu, Türkiye.; 3 Kartal Lutfi Kırdar City Hospital, Department of Neurology, Istanbul, Türkiye.

**Keywords:** Stroke, Collateral Circulation, Endovascular Procedures, Thrombectomy, Computed Tomography Angiography, Acidente Vascular Cerebral, Circulação Colateral, Procedimentos Endovasculares, Trombectomia, Angiografia por Tomografia Computadorizada

## Abstract

**Background** The relationship between collateral circulation and prognosis after endovascular treatment in anterior circulation strokes has been reported in many studies.

**Objective** In this study, we aimed to compare the predictive power of clinical outcome by comparing five different collateral scores that are frequently used.

**Methods** Among the patients who underwent endovascular treatment in our clinic between November 2019 and December 2021, patients with premorbid mRS < 3, intracranial ICA and/or MCA M1 occlusion, and a pre-procedural multiphase CTA examination were included in the study. Demographic, technical, and duration information about the procedure, major events after the procedure, and clinical outcomes at 3 months were recorded. The mCTA, Tan, Maas, Miteff, and rLMC collateral scores of the patients were evaluated.

**Results** Clinical outcome at 3 months were good in 37 of the 68 patients included in the study (mRS ≤ 2). Only the mCTA and rLMC collateral scores were statistically significantly higher in those with a good clinical outcome. Significant correlation with 3-month mRS was detected only in mCTA and rLMC scores. Although rLMC and mCTA collateral scores showed a statistically significant association with prognosis, they were not sufficient to be an independent predictor of prognosis.

**Conclusion** mCTA and rLMC were found to have the highest predictive power of clinical outcome and the highest correlation with the 3-month clinical outcome. Our study suggests that it would be beneficial to develop a new scoring system over multiphase CTA, which combines regional and temporal evaluation, which are the strengths of both collateral scoring.

## Resumo

**Antecedentes** A relação entre circulação colateral e prognóstico após tratamento endovascular em acidentes vasculares cerebrais de circulação anterior tem sido relatada em muitos estudos.

**Objetivo** Neste estudo, nosso objetivo foi comparar o poder preditivo do desfecho clínico comparando cinco escores colaterais diferentes que são frequentemente utilizados.

**Métodos** Entre os pacientes submetidos a tratamento endovascular em nossa clínica entre novembro de 2019 e dezembro de 2021, foram incluídos no estudo pacientes com mRS pré-mórbido < 3, oclusão intracraniana de ICA e/ou MCA M1 e exame de CTA multifásico pré-procedimento. Foram registradas informações demográficas, técnicas e de duração sobre o procedimento, eventos importantes após o procedimento e resultados clínicos em três meses. Foram avaliados os escores colaterais mCTA, Tan, Maas, Miteff e rLMC dos pacientes.

**Resultados** Os resultados clínicos aos três meses foram bons em 37 dos 68 pacientes incluídos no estudo (mRS ≤ 2). Apenas os escores colaterais mCTA e rLMC foram estatisticamente significativamente maiores naqueles com boa evolução clínica. Correlação significativa com mRS de três meses foi detectada apenas nos escores mCTA e rLMC. Embora os escores colaterais de rLMC e mCTA tenham mostrado uma associação estatisticamente significativa com o prognóstico, eles não foram suficientes para serem um preditor independente de prognóstico.

**Conclusão** Verificou-se que mCTA e rLMC têm o maior poder preditivo do resultado clínico e a maior correlação com o resultado clínico de três meses. Nosso estudo sugere que seria benéfico desenvolver um novo sistema de pontuação em vez de CTA multifásico, que combinasse avaliação regional e temporal, que são os pontos fortes de ambas as pontuações colaterais.

## INTRODUCTION

The efficacy of endovascular therapy (EVT) in anterior circulation strokes has been demonstrated by major randomized controlled trials and is recommended as Class I evidence level A in the guidelines. 1 The condition of collateral circulation is one of the most important factors that determines the prognosis of anterior circulation strokes. It has been reported in many studies that better collateral circulation is associated with smaller core infarcts, a lower neurological deficit, and a lower infarct growth rate. 2 3 4 5 Reports state that the collateral circulation not only makes the infarct area smaller but also reduces the irreversible damage to the blood vessels. The development of irreversible damage to the vessel, even if recanalization develops, causes extravasation of plasma and blood cells and hemorrhage. 6

Collateral circulation can be evaluated by magnetic resonance imaging (MRI), computed tomography (CT), and digital subtraction angiography (DSA) examinations. The gold standard The American Society of Interventional and Therapeutic Neuroradiology/Society of Interventional Radiology (ASITN/SIR) 7 collateral score is evaluated with DSA. Many scoring systems are used to evaluate collateral circulation over the CTA, but the number of studies comparing these scoring systems with each other is also very few. 8 9 There is no clear recommendation on which scoring method should be used. While most of the collateral scores are evaluated according to the vascular enhancement rate in the entire MCA area, 10 11 regional leptomeningeal collateral (rLMC) 12 evaluation is made regionally and a wider scoring range is offered. In this study, we evaluated the relationship between Tan, 11 Maas, 10 multiphase computed tomography angiography collateral score (mCTA), 13 rLMC, 12 and Miteff 14 collateral scores and clinical outcome, which are frequently used in clinical practice. We aimed to compare the ability of these scores to recognize good clinical outcomes.

## METHODS

**Figure 1:**
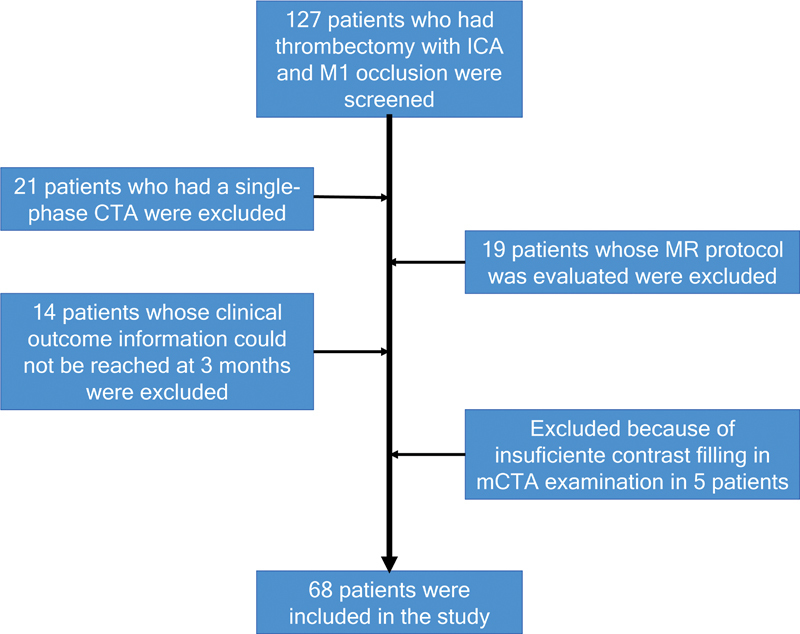
Flowchart.

After obtaining approval from the Bolu Abant Izzet Baysal University Clinical Research Ethics Committee (2023/34-21.02.2023), the files of patients who underwent EVT between November 2019 and December 2021 were scanned. Inclusion criteria for the study were: premorbid mRS < 3, presence of intracranial ICA and/or MCA M1 occlusion, and multiphase CTA examination before the procedure. Patients with distal occlusion, those who did not undergo CTA before the procedure, and those where clinical outcome information could not be obtained were excluded from the study. The clinical outcome at 3 months was obtained by a telephone conversation with the patients who did not attend the control examination. Patients with mRS 0-2 were considered good clinical outcomes. The files of 127 patients who underwent mechanical thrombectomy due to intracranial ICA and/or M1 occlusion were included. 21 patients were excluded from the study because they had single-phase CTA (sCTA), 19 were evaluated only with MRI, 14 could not reach the clinical outcome information at 3 month, and five had insufficient contrast filling despite mCTA examination. 68 patients were included in the study ( Figure 1 ). Patient' age, sex, admission NIHSS scores, and ASPECTS were noted. The data of the time, clinic, and the procedure, the technical data of the procedure, and the number of passes were noted. Post-procedure reperfusion categories, whether symptomatic intracerebral hemorrhage (sICH) developed or not were recorded.

### Imaging protocol

Non-contrast CT and CTA were performed using a multi-detector GE Revolition Evo 64 × 2 (General Electric Healthcare Chicago, USA). For non-contrast CT examination, images were acquired at 1 mm and 5 mm slice thickness. In CTA, 80-100 mL of nonionic contrast material was administered intravenously at a rate of 4.5 mL/sec with a Mallinckrodt automatic injector (Dublin, Ireland), and images with a slice thickness of 0.625 mm were obtained with the helical scanning technique. Maximum intensity projections (MIPs) images with a thickness of 5 mm were obtained in axial, coronal, and sagittal planes.

### Imaging analysis

Maas, Miteff, Tan, mCTA, rLMC collateral scores were calculated by retrospectively evaluating mCTA images by AO, an interventional neurologist with 6 years of experience, blinded to the clinical outcomes of the patients. For the mCTA examination, images were obtained in three phases as peak arterial phase, venous phase, and late venous phase in a total examination time of approximately 8 seconds. Maas, Miteff, Tan, and rLMC collateral scores were evaluated on arterial phase MIPs images obtained in the axial and coronal planes. The mCTA collateral score was evaluated on arterial, venous, and late venous phase images in the axial plane. Details of the collateral scoring systems used are given in Table 1 . The relationship between other data and collateral scores and good clinical outcome was evaluated, and it was estabilshed which collateral score was more successful in determining good clinical outcomes.

**Table 1:** CTA collateral scores evaluated in the study

**Maas** 10	**1:** There is no vessel opacification in the affected MCA territory **2:** Vessel opacification is lower compared with the opposite side **3:** Both MCA territories are opacified similarly **4:** Opacification of the affected MCA territory is higher compared with the opposite side **5:** Opacification of the affected MCA territory is exuberant
**Tan** 11	**0:** Absent collateral supply to the occluded MCA territory **1:** Collateral supply filling <50% but >0% of the occluded MCA territory **2:** Collateral supply filling >50% but <100% of the occluded MCA territory **3:** Collateral supply filling 100% of the occluded MCA territory
**rLMC** 12	rLMC score is based on scoring pial and lenticulostriate arteries (0, no; 1, less; 2, equal or more prominent compared with matching region in opposite hemisphere) in 6 ASPECTS regions (M1–6) plus anterior cerebral artery region and basal ganglia. Pial arteries in the Sylvian sulcus are scored 0, 2, or 4.
**mCTA** 13	**0:** No vessels visible in any phase within the ischemic vascular territory **1:** Only a few vessels are visible in any phase within the occluded vascular territory **2:** Either a delay of two phases in the filling of the peripheral vessels which decreased the prominence and extent or a one-phase delay and some ischemic regions with no vessels **3:** Either a delay of two phases in the filling of the peripheral vessels or the presence of a one-phase delay and significantly reduced number of vessels in the ischemic territory **4:** The presence of a delay of one phase in the filling of the peripheral vessels, with equal prominence and extent **5:** Either no delay or normal/increased prominence of the pial vessels/normal extent within the ischemic territory in the symptomatic hemisphere
**Miteff** 14	**1:** Only distal superficial MCA branches are reconstituted **2:** Some vessels in the Sylvian fissure show opacification **3:** The entire MCA distal to the occlusion is reconstituted

Abbreviations: mCTA, Multiphase computed tomography angiography collateral score; rLMC, regional leptomeningeal collateral score.

### Statistical analysis

Data were evaluated with the IBM SPSS 22 program. The Independent Sample *t* test was used to compare normally distributed independent groups, and the Mann Whitney U test was used to compare independent groups that did not show normal distribution. The Chi-squared test was used to evaluate categorical variables. The correlation of collateral scores with the third-month clinical outcome (evaluated as ordinal mRS) was evaluated by Spearman's rank correlation coefficient analysis. Univariate and multivariate binary logistic regression analysis was used to assess the association with prognosis.

## RESULTS

A total of 68 patients (40 females and 28 males), who met the inclusion criteria and had undergone EVT in our clinic, were included in the study. MCA M1 occlusion was present in 48 patients, intracranial ICA occlusion in 10 patients, and tandem occlusion in 10 patients. The patients were divided into two groups as good and poor clinical outcomes according to their 3-month clinical outcome. At 3 months, 37 patients had a good clinical outcome (mRS≤2), whereas, 31 patients had a poor clinical outcome (mRS≥3).

When demographic, radiological, and clinical data of patients with and without a good clinical outcome were compared, it was found that those with good clinical outcomes had significantly lower admission NIHSS scores and puncture recanalization times. ( *p =* 0.03, 0.04, respectively). The Tici 2b and above recanalization rate was significantly higher in patients with a good clinical outcome ( *p* = 0.035). When collateral scores were compared between patients with good and poor clinical outcomes, significant differences were observed only in rLMC and mCTA collateral scores ( *p =* 0.019 and 0.018, respectively) ( Table 2 ). When the relationship between 3-month mRS (ordinal mRS) with collateral scores was evaluated, a moderately significant correlation was found with mCTA and rLMC scores ( *p* 0.03, 0.028, respectively). There was no statistical significance with the Maas, Tan, and Miteff scores ( Table 3 ).

**Table 2:** Comparison of data of patients with good and poor clinical outcome

	Good clinical outcome (37)	Poor clinical outcome (31)	*p*
Age	71.16 ± 11.6	75.93 ± 10.89	0.088
Gender M/F	16/21	12/19	0.705
R/L	18/19	16/15	0.808
Occlusion site	M1: 28 ICA distal:3 Tandem: 6	M1: 20 ICA distal:7 Tandem: 4	0.243
Admission NIHSS	13.13 ± 4.42	15.83 ± 5.86	0.034*
ASPECTS	9 (7-10)	9 (5-10)	0.058
Onset-door time	214.81 ± 273.84	213.22 ± 247.84	0.980
Puncture-recanalization time	54.05 ± 38.09	73.58 ± 39.06	0.041*
Onset-recanalization time	346.67 ± 279.05	387.16 ± 253.68	0.537
Number of Passes	2.40 ± 1.51	3.09 ± 1.86	0.097
First pass recanalization	18	8	0.054
sICH	3	4	0.517
Successful recanalization (Tıcı≥2b)	35	23	0.035*
Tan	2 (1-3)	1 (1-3)	0.314
Miteff	2 (1-3)	2 (1-3)	0.119
Maas	2 (2-4)	2 (1-4)	0.308
rLMC	15 (4-19)	12(3-18)	0.019*
mCTA	3 (2-5)	2 (1-5)	0.018*

Abbreviations: F, Female; L, Left; M, Male; mCTA, multiphase computed tomography angiography collateral score; NIHSS, National Institutes of Health Stroke Scale; rLMC, regional leptomeningeal collateral; R, Right; sICH, symptomatic intracranial hemorrhage. Note: * *p* < 0.05.

**Table 3:** Correlation analysis with clinical outcome at 3rd months

		Age	NIHSS	ASPECTS	Tan	Maas	rLMC	mCTA	Onset-rec.	Puncture rec.
3rd month mRS	*r* *p*	0.240 0.048*	0.314 0.009*	-0.232 0.057	-0.158 0.197	-0.138 0.262	-0.266 0.028*	-0.263 0.030*	0.278 0.022*	0.328 0.006*

Abbreviations: mCTA, multiphase computed tomography angiography collateral score; NIHSS, National Institutes of Health Stroke Scale; rec, Recanalization; rLMC, regional leptomeningeal collateral. Note: * *p* < 0.05.

Univariate binary logistic regression analysis revealed that admission NIHSS, rLMC, and mCTA collateral scores were predictors of prognosis ( Table 4 ). In multivariate analysis with age, ASPECTS, NIHSS score, and collateral score systems (both mCTA and rLMC collateral scores) were not found to be sufficient as independent predictors of prognosis ( Tables 5 and 6 ).

**Table 4:** Evaluation of factors associated with prognosis by univariate logistic regression analysis

	B value	*p*
Age	0.039	0.092
Gender	-0.188	0.705
NIHSS	0.104	0.038*
ASPECT	-0.412	0.065
Tan	-0.326	0.309
Miteff	-0.546	0.120
Maas	-0.446	0.305
rLMC	-0.154	0.024*
mCTA	-0.547	0.022*

Abbreviations: mCTA, multiphase computed tomography angiography collateral score; rLMC, regional leptomeningeal collateral. Note: * *p* <0.05.

**Table 5:** Multivariate logistic regression analysis including mCTA collateral score and other variables

	B	*p*
Age	0.036	0.159
ASPECT	-0.341	0.161
NIHSS	0.053	0.349
mCTA	-0.321	0.225

Abbreviations: mCTA, multiphase computed tomography angiography collateral score; NIHSS, National Institutes of Health Stroke Scale.

**Table 6:** Multivariate logistic regression analysis including rLMC collateral score and other variables

	B	*p*
Age	0.041	0.116
ASPECTS	-0.330	0.177
NIHSS	0.053	0.338
rLMC	-0.109	0.147

Abbreviations: NIHSS, National Institutes of Health Stroke Scale; rLMC, regional leptomeningeal collateral.

## DISCUSSION

In this study, we found that mCTA and rLMC collateral scores were the most successful in predicting clinical outcomes, although their ability to recognize clinical outcomes was in the weak range, statistically. After arterial occlusion, collateral circulation tries to maintain perfusion in the vascular territory of the occluded segment. Good collateral circulation means less tissue is involved in irreversible damage, thus a smaller ischemic core. 3 It has been shown that collateral circulation can affect infarct volume and the success of the procedure independent of the time window. 2 Furthermore, it has been reported that even in cases longer than 24 hours, patients with good collateral circulation can benefit from endovascular treatment, and time is not an absolute criterion. 15 16 In a meta-analysis conducted by Quian et al. 17 with 5058 patients included, it was reported that patients with good collateral circulation had a better clinical outcome, a higher rate of successful reperfusion, and less symptomatic bleeding and mortality.

In the study conducted by Weiss et al. 9 the relationship between mTan, Maas, Miteff, Opercular index Score ratio (OISr) collateral scores, and good clinical outcome and CTP parameters were evaluated. In the ROC curve analysis, it was reported that the area under the curve was found in patients with the highest Miteff score, and the Maas score was significantly higher in those with a good clinical outcome and those who showed a moderate correlation with the 3-month mRS. With binary logistic regression analysis, the best result was obtained with the Maas score, and the highest sensitivity was obtained with the Maas score in the ROC curve analysis. In addition, CTP parameters were also evaluated in this study, and it was reported that the relationship of rCBV with good clinical outcome was more significant than collateral scores and that the Miteff score had the highest correlation with CBV. As a result, it has been reported that Maas and Miteff scores perform better in predicting good clinical outcome. In our study, however, no significant correlation was observed between Maas, Miteff, and Tan scores with 3-month mRS. We found that mCTA and rLMC scores, which were not evaluated by Weiss et al., significantly correlated with 3-month mRS. In a study by Seker et al. 8 in which Maas, Miteff, Tan, ASITN/SIR, and mCTA collateral scores were evaluated in relation to good clinical outcome, their ability to recognize good clinical outcome was found to be similar by ROC analysis. The correlation scores between 3-month mRS with Maas, Miteff, Tan, ASITN/SIR, and mCTA collateral scores were found to be low and interpreted as moderate correlation with 3-month clinical outcome. In conclusion, it has been reported that none of these collateral scores are sufficient to predict a good clinical outcome as an independent parameter. Similarly, in our study, no collateral scoring system alone was found to be an independent predictor of prognosis. In another study by Seker et al. 18 it was reported that the mismatch ratio and core infarct, ASPECTS collateral score, and ASITN/SIR collateral scores showed a good correlation, whereas Miteff and Christoforidis 19 scores did not. They reported that this might be because the ASPECTS collateral score can evaluate not only retrograde contrast filling but also vascular enhancement in the entire affected area with a wider scoring range. In our study, we found that the rLMC collateral score and mCTA scores, which evaluate the entire ICA territory by region similar to the ASPECTS collateral score, were the scores that best predicted the clinical outcome.

In the study by Gensicke et al. 20 in which Tan, regional leptomeningeal collateral score (rCS), and regional ASPECTS-based leptomeningeal collateral scores were evaluated on time-resolved CTA images, it was reported that all three collateral scores were associated with the clinical outcome at 3 months and that their predictive values were similar. Conversely, it was reported that the time of image acquisition independently affected the scoring in evaluations made over sCTA, and it was reported that the rCS collateral score had the strongest relationship with 3-month mRS.

In the study conducted by Peng et al. 21 it was reported that the mCTA collateral score was moderately correlated and that the rLMC score was lowly correlated with 3-month clinical outcome. In our study, both rLMC and mCTA scores were found to be correlated with clinical outcomes at 3 months. These findings show that mCTA, which provides a phasic evaluation of the collateral status, and rLMC, which provides a detailed regional evaluation, are more successful in determining the prognosis than scores with a simpler evaluation method.

With Maas, Miteff, and Tan collateral scores, collateral circulation is evaluated over a single phase and in a lesser range of scores without regional evaluation. Although the rLMC collateral score is also evaluated over a single phase, it provides a regional assessment in addition to these scorings, enabling the assessment of the collateral status within a higher score range and was, therefore, evaluated in our study. All of these scorings are made on a sCTA and do not provide information on the time of collateral circulation. It was reported by Wang et al. 22 that the duration of collateral circulation is also very important and that a short collateral circulation time is an independent predictor of good clinical outcomes. The mCTA score evaluates collateral circulation in three phases as arterial venous and late venous phases and provides information about the time of collateral circulation. Therefore, mCTA collateral scoring was also evaluated in our study. In our study, the ability to best recognize good clinical outcomes at 3 months and the best correlation with a 3-month outcome in mCTA and rLMC scores might also be due to the temporal and regional assessment they provide, respectively.

The major limitations of our study are that not all patients who underwent thrombectomy for various reasons mentioned in the method section could be included in the study, the clinical outcomes of some patients were obtained by telephone interview, and the study was retrospective. Another important limitation of our study was the relatively small number of patients, the fact that the collateral scoring was performed by a single person, and the inter-rater reliability was not evaluated. The advantages of our study are that there are few studies comparing collateral scores and also, studies comparing rLMC score with other collateral scores have not been performed before. In addition, our study reveals the need for collateral scoring that includes both regional evaluation and temporal evaluation, since the results of our study show that collateral scoring, which includes temporal and wider regional evaluation, is more successful.

In conclusion, although rLMC and mCTA scores had the best correlation with 3-month clinical outcome among CTA collateral scores, neither was sufficient to be an independent predictor of prognosis. Although it is difficult to recommend the use of any collateral score alone in patient selection from these findings, there is a need for multicenter studies with higher patient numbers in which mCTA and rLMC collateral scores and other parameters are evaluated together. Our study suggests that it would be beneficial to develop a new scoring system over multiphase CTA, which combines regional and temporal evaluation, which are the strengths of both collateral scorings.
